# The background occurrence of selected clinical conditions prior to the start of an extensive national vaccination program in Japan

**DOI:** 10.1371/journal.pone.0256379

**Published:** 2021-08-26

**Authors:** Tomotaka Sobue, Haruhisa Fukuda, Tetsuya Matsumoto, Bennett Lee, Shuhei Ito, Satoshi Iwata

**Affiliations:** 1 Division of Environmental Medicine and Population Science, Graduate School of Medicine, Osaka University, Osaka, Japan; 2 Department of Health Care Administration and Management, Kyushu University, Fukuoka, Japan; 3 Department of Infectious Diseases, International University of Health and Welfare, Narita, Japan; 4 Vaccine Medical Affairs, Pfizer Japan Inc., Tokyo, Japan; 5 Department of Infectious Diseases, National Cancer Center Hospital, Tokyo, Japan; 6 Department of Infectious Diseases, Keio University School of Medicine, Tokyo, Japan; Kaohsuing Medical University Hospital, TAIWAN

## Abstract

**Introduction:**

The COVID-19 pandemic caused by SARS-CoV-2 has now affected tens of millions of people globally. It is the hope that vaccines against SARS-CoV-2 will deliver a comprehensive solution to this global pandemic; however, this will require extensive national vaccination programs. Ultimately, clinical conditions and even sudden unexplained death will occur around the time of vaccination, thus a distinction needs to be made between events that are causally related to the vaccine or temporally related to vaccination. This study aimed to estimate the background occurrence of 43 clinical conditions in the Japanese population.

**Methods:**

A retrospective cohort study was conducted from 2013 to 2019 using data from two large healthcare claims databases (MDV and JMDC) in Japan. The estimated number of new cases and incidence were calculated based on the actual number of new cases identified in the databases. The PubMed and Ichushi-web databases, as well as grey literature such as guidelines and government statistics, were also searched to identify any publications related to incidence of these conditions in Japan.

**Results and conclusion:**

The estimates of the number of total cases and incidence were similar for the MDV and JMDC databases for some diseases. In addition, some estimates were similar to those in the scientific literature. For example, from the MDV and JMDC databases, estimates of incidence of confirmed Bell’s palsy in 2019 were 41.7 and 47.9 cases per 100,000 population per year, respectively. These estimates were of the same order from the scientific publication. Determining whether clinical conditions occurring around the time of vaccination are causally or only temporally related to vaccination will be critical for public health decision makers as well as for the general public. Comparison of background occurrence at the population level may provide some additional objective evidence for the evaluation of temporality or causality.

## Introduction

Coronavirus disease 2019 (Covid-19) caused by the SARS-CoV-2 virus has now affected tens of millions of people globally. In December 2019, the first clinical case was reported in Wuhan, China. On 11 March 2020 the WHO declared a pandemic [1]. At the time of writing, the WHO estimates that over 181 million cases have been confirmed and over 3 million deaths have occurred globally [2]. The enormous morbidity and mortality due to COVID-19 has severely strained the healthcare resources of affected countries, deeply affected the social well-being of their citizens and devastated their economies. The repeated attempts at controlling the spread of SARS-CoV-2 through social distancing, frequent hand washing and mask wearing coupled with case identification, quarantining and population “lockdown” measures have been unsuccessful in completely halting the spread of the disease. It is the hope therefore that vaccines against SARS-CoV-2 will be the public health measure that delivers a comprehensive solution to this global pandemic.

It is expected however that vaccination programs against SARS-CoV-2 will require high vaccination coverage to protect the most vulnerable individuals and to provide, if transmission can be interrupted through vaccination, “herd protection” that will halt further spread of this virus [3, 4]. Attaining such high vaccination coverage in the population in as short a time as possible will require unprecedented mobilization of healthcare resources to deliver available vaccines and unwavering population willingness to accept the safety profile and personal and public health value of vaccination.

Local and systemic adverse reactions are associated with all vaccines in use today. However, the benefit of vaccination far outweighs the impact of these adverse reactions and this is generally well accepted by society [5]. However, in the context of extensive national vaccination programs that will include many millions of individuals, there is a certainty that severe clinical conditions and even sudden unexplained death not causally related to vaccination but only temporally related to it will occur [6]. These events have a clear risk of damaging trust in the vaccination program, reinforcing vaccination hesitancy and reducing the likelihood of attaining the high vaccination coverage needed to combat this global pandemic [4]. Even though evaluations of new vaccines are conducted with well-designed randomized control trials, these may not be able to identify very rare adverse events. The purpose of this study is to collate data on the occurrence of selected clinical conditions in Japan which may be observed to have a temporal relationship with vaccination against COVID-19. Each occurrence of a clinical condition deemed to be potentially causally related to vaccination needs to be investigated thoroughly within the obligatory Post Marketing Surveillance (PMS) system that has been implemented in collaboration with the Ministry of Health, Labour and Welfare and the Regulatory Authorities in Japan [7]. The purpose of this study is not to replace this PMS but to provide some evidence that outside of the vaccination program against COVID-19 these clinical conditions and sudden unexplained deaths do occur, giving the authorities the opportunity and time to thoroughly and appropriately investigate the possibility of a causal relationship with vaccination.

To our knowledge, this study is the first of its kind in Japan to evaluate the occurrence of 43 selected clinical conditions prior to the start of an extensive national vaccination program. It builds on the precedent set by other studies of a similar nature. The study by Black et al. (2009) estimated the background rates of disease for the assessment of vaccine safety during mass vaccination programs against pandemic H1N1 influenza. The authors concluded that awareness of the background rates of possible adverse events would be a crucial part of assessment of possible vaccine safety concerns and would help to separate legitimate safety concerns from events that are temporally associated with, but not caused by, vaccination [8]. Similarly following the H1N1 vaccination program in China, Wang et al. (2013) estimated the background occurrence of clinical events in China during the H1N1 influenza pandemic vaccination program and concluded that even for rare concurrent events, a large number of events can be expected in a short period because of the large population targeted for vaccination [9]. Since the H1N1 pandemic is the most recent public health event of its kind before the present SARS-CoV-2 pandemic, we used the studies by Black S et al. and Wang Y et al. to inform the need for and the design of this study. Our study sought to estimate the number of new cases for 43 clinical conditions which occurred in Japan between 2015–2019 using two different large-scale Japanese healthcare claims databases and to validate these findings with data from publications in peer-reviewed journals and other appropriate sources.

## Methods

### Study design

This retrospective database study was conducted from 1 January 2013 to 31 December 2019 using two large-scale healthcare claims databases, the Japan Medical Data Center (JMDC) and the Medical Data Vision Co. Ltd. (MDV). According to the joint guidelines (latest revision 23 March 2021) of MEXT (Ministry of Education, Culture, Sports, Science and Technology), MHLW (Ministry of Health, Labour and Welfare) and METI (Ministry of Economy, Trade and Industry), the current study does not require any ethics committee approval because the data derived from the MDV and JMDC databases used in the study only include anonymized de-identified/de-linked information. In this study, 43 different clinical conditions which are defined by the 10th revision of the International Statistical Classification of Diseases and Related Health Problems (ICD-10) codes were selected based on (1) WHO publications, (2) the studies conducted by Black et al. and Wang Y et al. [8, 9], and (3) clinical importance and frequency advised by medical experts. A detailed list of all 43 conditions and their specific ICD-10 codes are shown in Table 1.

**Table 1:** ICD-10 codes used to identify clinical condition outcomes.

Clinical condition outcome	ICD-10 codes
Acute Disseminated Encephalomyelitis	G040
Acute Transverse Myelitis	G37 or G373
Amyotrophic Lateral Sclerosis	G122
Anaphylaxis / Shock	T782, T805, T886
Any Death / Sudden Unexplained Death	R95, R96, R98, R99
Ataxia	R270, R278
Bell’s Palsy	G510
Brachial Neuritis	G540
Hereditary Ataxia	G11
Degenerative disease of nervous system, unspecified	G319
Chronic Fatigue Syndrome	G933
Chronic Inflammatory Disseminated Polyneuropathy	G618
Chronic Urticaria	L508
Complex Regional Pain Syndrome	M8900, G564
Deltoid Bursitis	M755
Drug-induced Interstitial Pneumonia	J702, J704
Encephalitis	A170, A178, A321, A398, A504, A521, A811, A830, A831, A832, A833, A834, A835, A840, A841, A849, A850, A851, A852, A858, A86, A872, A922, B004, B011, B020, B050, B060, B258, B262, B569, B574, B582, B602, B608, B832, B941, C809, F071, G040, G048, G049, G09, G213, G361, G370, G405, G610, M321
Encephalopathy	A080, A081, A810, A812, B082, B220, B348, E063, E161, E512, E52, E569, E725, E752, E870, F011, F058, F069, F072, F078, F107, G312, G318, G319, G404, G408, G459, G92, G931, G934, G938, I672, I673, I674, I678, J118, K729, K868, N185, O211, P112, P916, Q000, Q041, Q042, Q043, Q045, Q046, Q878, T58
Erythema Nodosum	L52
Guillain-Barré Syndrome	G610
Hepatitis (Autoimmune)	K754
Hypercoagulable States (Autoimmune)	D686
Hypersensitivity Pneumonitis	J679
Allergic Purpura	D690
Idiopathic Thrombocytopenic Purpura	D693
Interstitial Pneumonia	J841, J849
Juvenile Idiopathic Arthritis	M08
Meningitis	A010, A022, A170, A203, A228, A279, A321, A390, A504, A514, A521, A548, A692, A809, A870, A871, A872, A879, B003, B010, B021, B051, B060, B261, B279, B375, B384, B451, B49, B569, B574, B602, B832, B900, C793, G000, G001, G002, G003, G008, G009, G030, G031, G032, G039, G042, G049, G09, M321, T798, T814
Multiple Sclerosis	G35
Myocardial Infarction	I21
Myocarditis	I408, I409, I514
Neuromyelitis Optica	G360
Optic Neuritis	H46
Overlapping (Encephalitis/Meningitis)	A170, A321, A504, A521, A872, B060, B569, B574, B602, B832, G049, G09, M321
Polyarteritis Nodosa	M300, M308
Preterm Labor	O60
Pulmonary Embolism	I260, I269
Reactive Arthritis	M0239, M0299
Rheumatoid Arthritis	M05, M06
Seizure	G568 G40, G41
Spontaneous Abortion	O031, O032, O033, O034, O036, O037, O038, O039
Stroke	I60, I61, I62, I63, I64, I66, I679
Systemic Lupus Erythematous	M32

### Data sources

Two major healthcare claim databases in Japan, JMDC and MDV, were used for this study. The JMDC database contains claims data of inpatients and outpatients from the Japanese union-managed health insurance system, comprising 10 health insurance societies with a present dataset of 7.3 million individuals as of April 2020 [10]. The JMDC database includes workers mostly aged < 65 years employed by mid- to large-sized companies and their dependents, and excludes individuals aged ≥ 75 years.

The MDV database contains health insurance claims for inpatients and outpatients primarily from Diagnosis Procedure Combination (DPC) hospitals [11]. DPC is the claims-based payment system used by larger hospitals in Japan. The number of DPC hospitals providing data to the MDV database has increased over the years since its inception. This increase in participation was taken into consideration in the extrapolation of the data from the MDV database to all of Japan. The number of DPC hospitals in Japan and the number providing data to the MDV database in each period in this study are included in Table 2. Each hospital had to be including data in the database for at least 2 years for its data to be included in the evaluation of the occurrence of clinical conditions. The rationale for this is described in the following section.

**Table 2:** Number of DPC hospitals in Japan and included in MDV database by year.

Year	Period the hospital needed to have provided data to include in the study	In MDV database	In Japan	Percentage of DPC hospitals providing data
2019	January 2017 to December 2019	341	1,667	20%
2018	January 2016 to December 2018	290	1,580	18%
2017	January 2015 to December 2017	253	1,585	16%
2016	January 2014 to December 2016	175	1,496	12%
2015	January 2013 to December 2015	124	1,505	8%

### Estimation of incidence

In this study, the number of new cases for each clinical condition was counted from the database according to the following two definitions: (a) Definition 1 included patients with a confirmed diagnosis or suspected diagnosis within a 24-month look-back period and (b) Definition 2 included patients with a confirmed diagnosis only within a 24-month look-back period. A period of 24 months prior to the observed diagnosis was used to identify any subjects that had a previous diagnosis in the study period to separate incident events (i.e., new events) from repeat episodes of the same diagnosis (i.e., prevalent events). For example, for all diagnoses of a clinical condition observed in 2019 we ensured that the same diagnosis was not included in the 24 months prior to the 2019 diagnosis. This approach greatly increased the likelihood that only new cases of the clinical condition were being included for a given year in both the JMDC and MDV databases.

We calculated the estimated incidence of each clinical condition in the Japanese population based on the actual number of new cases identified in the database analysis. Regarding the JMDC database, the age-specific incidence for each clinical condition in a given year was based on the observations of new cases in each age group and the number of individuals in that age group registered in the health insurance system and covered by the database in that year.


Incidence(JMDC)=#Newcases(JMDC)/#healthinsuranceholders(JMDC)

For the MDV database, the corresponding calculation of the incidence for each clinical condition was conducted based on the proportion of DPC hospitals included in the MDV database, and the number of DPC hospitals in Japan for each year. The number of new cases observed in a particular year and the population in Japan for that year came from the Statistics Bureau of Japan.


Incidence(MDV)=#Newcases(MDV)x(#DPChospitals(Japan)/#DPChospitals(MDV))/populationfromthenationalcensus

The definition of each clinical condition was based on the ICD-10 codes in both databases, but ICD-10 codes do not specify disease severity. Some patients may have had two or more competing disease events at the same time, and every event was counted separately in the analysis of each disease condition. The MDV database consists of data from DPC hospitals and does not include any data from non-DPC hospitals. Therefore, the number of patients in non-DPC hospitals was assumed to be zero in this analysis for the MDV database.

The results of the analyses for 14 of the 43 clinical conditions listed below are included in the main report. The results for the remaining clinical conditions in this study are included in the accompanying S1 Table. The 14 clinical conditions are:

(1) Bell’s palsy, (2) Guillain-Barre syndrome, (3) Optic Neuritis, (4) Idiopathic Thrombocytopenic Purpura, (5) Multiple Sclerosis, (6) Complex Regional Pain Syndrome, (7) Hypercoagulable States (Autoimmune), (8) Any Death / Sudden Unexplained Death, (9) Acute Transverse Myelitis, (10) Allergic Purpura, (11) Anaphylaxis/Shock, (12) Seizure, (13) Preterm Labor and (14) Spontaneous Abortion.

The 14 conditions have been roughly sorted according to the following conditions: 1–8 are those for which estimated incidence increases by age, 9–12 are those for which estimated incidence is similar among the age groups, and 13–14 are those related to pregnant women.

### Literature search

This study performed a comprehensive search of the Japanese medical literature to collate and analyze the available data that describe the background incidence of underlying clinical conditions in Japan. Published papers from PubMed (https://pubmed.ncbi.nlm.nih.gov/) and Ichushi-web (https://www.jamas.or.jp/english/) were searched and retrieved. For the literature searches no date range was specified and results up until the last date of searching (17 March 2021) were considered. Inclusion and exclusion criteria for the literature searches is shown in S2 Table as a PICOST (Population, Intervention, Comparator, Outcomes, Study type and Timeframe) table. An example search strategy in PubMed for the clinical condition encephalitis is shown in S3 Table. Priority was given to the most recent publications and to those literature that focused on age-specific and gender-specific incidence. When Japanese literature was not available global literature was searched to identify those conditions that included incidence. Grey literature such as guidelines and government statistics were also reviewed. In combination with the diagnostic keywords, terms such as incidence, prevalence and epidemiology were also used when searching the literature. Summary information and references for the studies included in the literature search results are shown at the end of each of the 14 clinical condition tables in the main text and in S1 Table.

## Results

The background rate of occurrence of the 43 identified clinical conditions in Japan were estimated through retrospective database analysis. The results for the 14 target clinical conditions based on the analyses using the MDV and JMDC databases for the year 2019 are described below. For the literature search, including grey literature, 299 English publications and 130 Japanese publications were manually reviewed. All the clinical conditions identified in this study and their occurrence rates for each consecutive year from 2015 to 2019 are presented in the S1 Table.

The estimated occurrence of new cases “N” and the incidence “per 100,000 population per year” of confirmed or suspected diagnoses (Definition 1) and confirmed diagnoses (Definition 2) for each of the 14 clinical conditions reported in Japan in the year 2019 are shown in Tables 3–16, as follows.

**Table 3:** Estimated incidence of Bell’s palsy.

Bell’s Palsy	MDV				JMDC			
	New cases with confirmed or suspected diagnosis in 2019		New cases with confirmed diagnosis in 2019		New cases with confirmed or suspected diagnosis in 2019		New cases with confirmed diagnosis in 2019	
	N	Incidence^#^	N	Incidence^#^	N	Incidence^#^	N	Incidence^#^
Total cases	53,163	42.1	52,596	41.7	69,723	54.0	61,830	47.9
**Gender**								
Male	27,220	44.3	26,995	44.0	34,582	55.1	32,674	52.0
Female	25,944	40.1	25,601	39.5	35,141	53.0	29,156	44.0
**Age (years old)**								
0–11	1,061	8.8	1,051	8.8	846	6.9	725	5.9
12–17	1,046	15.9	1,041	15.8	1,070	16.0	910	13.6
18–24	1,501	17.0	1,501	17.0	1,782	20.1	1,689	19.1
25–44	9,802	33.5	9,694	33.1	14,582	48.6	13,518	45.1
45–64	15,990	47.6	15,854	47.2	22,938	67.2	21,493	62.9
65–74	10,481	60.3	10,315	59.3	28,505	76.8	23,495	63.3
75-*	12,275	66.4	12,138	65.6				
**Literature review data**								
A database study which included healthcare databases in Denmark, Italy, Spain and the UK between 2003 and 2014 reported that the incidence rate of Bell’s palsy was 23.8 per 100,000 person-years [13].								

* As patients above 75 years of age were few, they were combined with the age group 65–74 years in the JMDC database.

^#^ Estimated incidence per 100,000 population per year.

**Table 4:** Estimated incidence of Guillain–Barre Syndrome.

Guillain-Barré Syndrome	MDV				JMDC			
	New cases with confirmed or suspected diagnosis in 2019		New cases with confirmed diagnosis in 2019		New cases with confirmed or suspected diagnosis in 2019		New cases with confirmed diagnosis in 2019	
	N	Incidence^#^	N	Incidence^#^	N	Incidence^#^	N	Incidence^#^
Total cases	7,264	5.8	4,810	3.8	12,870	10.0	4,754	3.7
**Gender**								
Male	4,014	6.5	2,635	4.3	7,752	12.3	3,382	5.4
Female	3,251	5.0	2,175	3.4	5,118	7.7	1,372	2.1
**Age (years old)**								
0–11	147	1.2	112	0.9	378	3.1	155	1.3
12–17	308	4.7	220	3.3	774	11.5	197	2.9
18–24	376	4.3	259	2.9	880	9.9	243	2.7
25–44	1,335	4.6	968	3.3	3,494	11.6	1,238	4.1
45–64	2,009	6.0	1,335	4.0	4,188	12.3	1,658	4.9
65–74	1,403	8.1	836	4.8	3,156	8.5	1,263	3.4
75-*	1,579	8.5	1,007	5.4				
**Literature review data**								
A survey conducted in Japan between 1993 and 1998 showed that the incidence rate of Guillain-Barré Syndrome was estimated to be 1.15 per 100,000 population per year [15, 16]. An epidemiological survey conducted in Tokushima and Kochi, Japan, between 2006 and 2015 showed that the incidence rate of Guillain-Barré syndrome was 0.42 cases per 100,000 person-years and that of Fisher syndrome was 0.22 cases per 100,000 person-years [17]. A meta-analysis of 16 studies conducted in North America and Europe showed an exponential increase in Guillain-Barré syndrome incidence from 0.62 to 2.66 per 100,000 person-years across all age groups [18].								

* As patients above 75 years of age were few, they were combined with the age group 65–74 years in the JMDC database.

^#^ Estimated incidence per 100,000 population per year.

**Table 5:** Estimated incidence of optic neuritis.

Optic Neuritis	MDV				JMDC			
	New cases with confirmed or suspected diagnosis in 2019		New cases with confirmed diagnosis in 2019		New cases with confirmed or suspected diagnosis in 2019		New cases with confirmed diagnosis in 2019	
	N	Incidence^#^	N	Incidence^#^	N	Incidence^#^	N	Incidence^#^
Total cases	16,352	13.0	11,297	9.0	96,145	74.5	32,082	24.9
**Gender**								
Male	7,392	12.0	5,206	8.5	42,460	67.6	13,952	22.2
Female	8,961	13.8	6,091	9.4	53,685	81.0	18,130	27.4
**Age (years old)**								
0–11	362	3.0	210	1.8	7,430	60.8	1,379	11.3
12–17	499	7.6	396	6.0	5,424	80.9	1,109	16.5
18–24	460	5.2	337	3.8	4,243	47.9	1,151	13.0
25–44	2,698	9.2	1,980	6.8	15,401	51.3	4,984	16.6
45–64	4,375	13.0	3,138	9.3	24,853	72.8	8,867	26.0
65–74	3,354	19.3	2,317	13.3	38,794	104.6	14,592	39.3
75-*	4,273	23.1	2,713	14.7				
**Literature review data**								
A nationwide survey in Japan between 1992 and 1993 reported that the incidence rate of idiopathic optic neuritis was 1.03 per 100,000 population per year, and that among adults was 1.62. The male-female ratio of the incidence was 1:1.22, and 65.9% of the incidence was among patients 14–55 years old [20]. A cohort study conducted in the United Kingdom between 1995 and 2019 showed that the incidence across 22 years was stable at 3.7 (95% CI, 3.6–3.9) per 100,000 person-years [21].								

* As patients above 75 years of age were few, they were combined with the age group 65–74 years in the JMDC database.

^#^ Estimated incidence per 100,000 population per year.

**Table 6:** Estimated incidence of idiopathic thrombocytopenic purpura.

Idiopathic Thrombocytopenic Purpura	MDV				JMDC			
	New cases with confirmed or suspected diagnosis in 2019		New cases with confirmed diagnosis in 2019		New cases with confirmed or suspected diagnosis in 2019		New cases with confirmed diagnosis in 2019	
	N	Incidence^#^	N	Incidence^#^	N	Incidence^#^	N	Incidence^#^
Total cases	20,063	15.9	16,851	13.4	23,551	18.3	9,421	7.3
**Gender**								
Male	9,234	15.0	7,655	12.5	10,217	16.3	4,954	7.9
Female	10,828	16.7	9,195	14.2	13,334	20.1	4,467	6.7
**Age (years old)**								
0–11	1,437	12.0	1,281	10.7	1,368	11.2	668	5.5
12–17	303	4.6	284	4.3	569	8.5	265	4.0
18–24	445	5.0	416	4.7	691	7.8	291	3.3
25–44	2,048	7.0	1,755	6.0	4,441	14.8	1,601	5.3
45–64	3,710	11.0	3,065	9.1	6,103	17.9	3,202	9.4
65–74	3,696	21.2	3,036	17.5	10,379	28.0	3,394	9.1
75-*	7,963	43.1	6,629	35.9				
**Literature review data**								
A study using a database of the Ministry of Health, Labour and Welfare in Japan between 2004 and 2007 reported that the incidence rate of ITP was 2.16 per 100,000 population per year, and that in men was 1.72 and in women was 2.58 [23].								

* As patients above 75 years of age were few, they were combined with the age group 65–74 years in the JMDC database.

^#^ Estimated incidence per 100,000 population per year.

**Table 7:** Estimated incidence of multiple sclerosis.

Multiple Sclerosis	MDV				JMDC			
	New cases with confirmed or suspected diagnosis in 2019		New cases with confirmed diagnosis in 2019		New cases with confirmed or suspected diagnosis in 2019		New cases with confirmed diagnosis in 2019	
	N	Incidence^#^	N	Incidence^#^	N	Incidence^#^	N	Incidence^#^
Total cases	9,948	7.9	4,634	3.7	22,660	17.6	3,571	2.8
**Gender**								
Male	4,131	6.7	1,657	2.7	9,544	15.2	1,639	2.6
Female	5,817	9.0	2,977	4.6	13,116	19.8	1,932	2.9
**Age (years old)**								
0–11	117	1.0	44	0.4	224	1.8	17	0.1
12–17	328	5.0	142	2.1	627	9.4	71	1.1
18–24	650	7.4	308	3.5	1,098	12.4	417	4.7
25–44	2,977	10.2	1,618	5.5	5,625	18.7	1,162	3.9
45–64	3,031	9.0	1,564	4.7	6,150	18.0	1,459	4.3
65–74	1,442	8.3	499	2.9	8,936	24.1	445	1.2
75-*	1,266	6.8	411	2.2				
**Literature review data**								
A nationwide survey in Japan reported that the prevalence was 7.7 per 100,000 population and the number of patients was estimated to be 9,900 in 2004 in Japan, including Neuromyelitis optica (NMO). The ratio of males-females was 1:2.9 and the peak in age was in 25–29 year-olds [25]. An epidemiologic surveillance conducted in Tokachi, Hokkaido, Japan, reported that the prevalence of multiple sclerosis was 16.2 per 100,000 population (7.7 for males and 24.0 for females) in 2011 [26].								

* As patients above 75 years of age were few, they were combined with the age group 65–74 years in the JMDC database.

^#^ Estimated incidence per 100,000 population per year.

**Table 8:** Estimated incidence of complex regional pain syndrome.

Complex Regional Pain Syndrome	MDV				JMDC			
	New cases with confirmed or suspected diagnosis in 2019		New cases with confirmed diagnosis in 2019		New cases with confirmed or suspected diagnosis in 2019		New cases with confirmed diagnosis in 2019	
	N	Incidence^#^	N	Incidence^#^	N	Incidence^#^	N	Incidence^#^
Total cases	3,828	3.0	3,740	3.0	7,031	5.4	6,750	5.2
**Gender**								
Male	1,447	2.4	1,398	2.3	2,391	3.8	2,347	3.7
Female	2,381	3.7	2,342	3.6	4,640	7.0	4,403	6.6
**Age (years old)**								
0–11	34	0.3	29	0.2	69	0.6	69	0.6
12–17	54	0.8	49	0.7	180	2.7	180	2.7
18–24	98	1.1	93	1.1	38	0.4	38	0.4
25–44	401	1.4	386	1.3	981	3.3	964	3.2
45–64	1,354	4.0	1,325	3.9	2,723	8.0	2,459	7.2
65–74	929	5.3	919	5.3	3,040	8.2	3,040	8.2
75-*	880	4.8	860	4.7				
**Literature review data**								
A comprehensive review based on data from several countries revealed a broad incidence of 0.82–26.2 per 100,000 population per year [28].								

* As patients above 75 years of age were few, they were combined with the age group 65–74 years in the JMDC database.

^#^ Estimated incidence per 100,000 population per year.

**Table 9:** Estimated incidence of Hypercoagulable States (Autoimmune).

Hypercoagulable States (Autoimmune)	MDV				JMDC			
	New cases with confirmed or suspected diagnosis in 2019		New cases with confirmed diagnosis in 2019		New cases with confirmed or suspected diagnosis in 2019		New cases with confirmed diagnosis in 2019	
	N	Incidence^#^	N	Incidence^#^	N	Incidence^#^	N	Incidence^#^
Total cases	33,477	26.5	7,308	5.8	88,269	68.4	13,660	10.6
**Gender**								
Male	10,256	16.7	1,745	2.8	25,472	40.6	2,365	3.8
Female	23,221	35.9	5,563	8.6	62,797	94.8	11,295	17.0
**Age (years old)**								
0–11	259	2.2	64	0.5	704	5.8	69	0.6
12–17	538	8.2	108	1.6	1,260	18.8	160	2.4
18–24	1,300	14.7	279	3.2	3,462	39.1	629	7.1
25–44	9,171	31.3	2,708	9.3	29,192	97.3	7,559	25.2
45–64	8,941	26.6	1,760	5.2	23,141	67.7	2,503	7.3
65–74	5,891	33.9	1,056	6.1	30,510	82.2	2,740	7.4
75-*	6,883	37.2	1,247	6.7				
**Literature review data**								
A population-based study in the USA reported that the incidence rate of antiphospholipid syndrome was 2.1 per 100,000 population per year among adults aged ≥ 18 years [30].								

* As patients above 75 years of age were few, they were combined with the age group 65–74 years in the JMDC database.

^#^ Estimated incidence per 100,000 population per year.

**Table 10:** Estimated incidence of Any Death/Sudden Unexplained Death.

Any Death / Sudden Unexplained Death	MDV				JMDC			
	New cases with confirmed or suspected diagnosis in 2019		New cases with confirmed diagnosis in 2019		New cases with confirmed or suspected diagnosis in 2019		New cases with confirmed diagnosis in 2019	
	N	Incidence^#^	N	Incidence^#^	N	Incidence^#^	N	Incidence^#^
Total cases	494	0.4	484	0.4	71	0.1	52	0.0
**Gender**								
Male	298	0.5	288	0.5	19	0.0	0	0.0
Female	196	0.3	196	0.3	52	0.1	52	0.1
**Age (years old)**								
0–11	34	0.3	24	0.2	0	0.0	0	0.0
12–17	0	0.0	0	0.0	0	0.0	0	0.0
18–24	0	0.0	0	0.0	0	0.0	0	0.0
25–44	15	0.1	15	0.1	19	0.1	0	0.0
45–64	64	0.2	64	0.2	52	0.2	52	0.2
65–74	73	0.4	73	0.4	0	0.0	0	0.0
75-*	308	1.7	308	1.7				
**Literature review data**								
According to the data of the Utstein registry of the Fire and Disaster Management Agency, the number of the sudden cardiac death was 78,884 in Japan in 2019 (The incidence rate is estimated to be 62 per 100,000 population per year.) [32]. In a study from Okinawa, Japan, for the period 1 January 1992 to 31 December 1994, the incidence of sudden unexpected death was observed to be 37 per 100,000 population per year (51 for males aged 20–74 years, and 23 for females aged 20–74 years) [31].								

* As patients above 75 years of age were few, they were combined with the age group 65–74 years in the JMDC database.

^#^ Estimated incidence per 100,000 population per year.

**Table 11:** Estimated incidence of Acute Transverse Myelitis.

Acute Transverse Myelitis	MDV				JMDC			
	New cases with confirmed or suspected diagnosis in 2019		New cases with confirmed diagnosis in 2019		New cases with confirmed or suspected diagnosis in 2019		New cases with confirmed diagnosis in 2019	
	N	Incidence^#^	N	Incidence^#^	N	Incidence^#^	N	Incidence^#^
Total cases	953	0.8	753	0.6	2,104	1.6	644	0.5
**Gender**								
Male	494	0.8	420	0.7	747	1.2	415	0.7
Female	460	0.7	332	0.5	1,357	2.0	229	0.3
**Age (years old)**								
0–11	49	0.4	29	0.2	290	2.4	137	1.1
12–17	20	0.3	10	0.1	88	1.3	52	0.8
18–24	20	0.2	15	0.2	179	2.0	47	0.5
25–44	181	0.6	142	0.5	286	1.0	106	0.4
45–64	284	0.8	230	0.7	559	1.6	302	0.9
65–74	200	1.2	156	0.9	702	1.9	0	0.0
75-*	191	1.0	161	0.9				
**Literature review data**								
A survey in Fukuoka, Japan, in September 1998 to August 2003 reported that the incidence among 2–13 year old children was 0.11 per 100,000 person-years of observation [35]. A study reviewing articles published between 1981 and 2009 showed that the incidence of acute transverse myelitis was between 1.34 and 4.6 per million per year with bimodal peaks between ages 10–19 and 30–39 years [36].								

* As patients above 75 years of age were few, they were combined with the age group 65–74 years in the JMDC database.

^#^ Estimated incidence per 100,000 population per year.

**Table 12:** Estimated incidence of Allergic Purpura.

Allergic Purpura	MDV				JMDC			
	New cases with confirmed or suspected diagnosis in 2019		New cases with confirmed diagnosis in 2019		New cases with confirmed or suspected diagnosis in 2019		New cases with confirmed diagnosis in 2019	
	N	Incidence^#^	N	Incidence^#^	N	Incidence^#^	N	Incidence^#^
Total cases	17,613	14.0	15,604	12.4	35,703	27.7	23,831	18.5
**Gender**								
Male	8,443	13.7	7,582	12.3	13,657	21.8	9,348	14.9
Female	9,171	14.2	8,022	12.4	22,046	33.3	14,483	21.9
**Age (years old)**								
0–11	7,338	61.1	7,230	60.2	10,908	89.2	8,357	68.3
12–17	753	11.4	684	10.4	1,985	29.6	1,150	17.2
18–24	577	6.5	533	6.0	1,196	13.5	859	9.7
25–44	2,146	7.3	1,902	6.5	5,624	18.7	3,888	13.0
45–64	2,361	7.0	1,848	5.5	6,740	19.7	4,250	12.4
65–74	1,843	10.6	1,418	8.2	9,250	24.9	5,327	14.4
75-*	2,435	13.2	1,867	10.1				
**Literature review data**								
A population-based study in the UK between 1996 and 1999 reported that the incidence rate of Henoch-Schönlein purpura (HSP) was 20.4 per 100,000 population per year in children < 17 years of age, with a peak incidence of 70.3 in children between the ages of 4 and 6 years [38]. A database study conducted in Taiwan between 1999 and 2002 reported that the incidence rate of HSP was 12.9 per 100,000 population per year in children < 17 years of age, with a peak incidence of 26.6 at the age of 5 years and 27.9 at the age of 6 years [39]. A database study conducted in Korea between 2006 and 2015 reported that the incidence of HSP was 55.9 per 100,000 population per year in children < 18 years of age, with a peak incidence of 121.6 at the age of 5 years [40].								

* As patients above 75 years of age were few, they were combined with the age group 65–74 years in the JMDC database.

^#^ Estimated incidence per 100,000 population per year.

**Table 13:** Estimated incidence of Anaphylaxis/Shock.

Anaphylaxis/Shock	MDV				JMDC			
	New cases with confirmed or suspected diagnosis in 2019		New cases with confirmed diagnosis in 2019		New cases with confirmed or suspected diagnosis in 2019		New cases with confirmed diagnosis in 2019	
	N	Incidence^#^	N	Incidence^#^	N	Incidence^#^	N	Incidence^#^
Total cases	66,836	53.0	66,255	52.5	95,695	74.2	87,847	68.1
**Gender**								
Male	34,064	55.5	33,848	55.1	46,646	74.3	44,035	70.1
Female	32,773	50.6	32,406	50.0	49,049	74.0	43,812	66.1
**Age (years old)**								
0–11	14,348	119.5	14,328	119.4	17,644	144.3	16,445	134.5
12–17	3,691	55.9	3,676	55.7	7,293	108.8	6,791	101.3
18–24	4,018	45.6	3,984	45.2	5,563	62.8	4,965	56.0
25–44	11,879	40.6	11,767	40.2	17,283	57.6	15,677	52.3
45–64	14,446	43.0	14,309	42.6	22,598	66.2	20,429	59.8
65–74	9,645	55.5	9,547	54.9	25,314	68.2	23,540	63.5
75-*	7,978	43.1	7,812	42.2				
**Literature review data**								
A survey conducted by the Ministry of Education, Culture, Sports, Science and Technology in Japan in 2013 reported that the proportion of students with a history of anaphylaxis was 0.6% in elementary school students, 0.4% in junior high school students and 0.3% in high school students [42].								

* As patients above 75 years of age were few, they were combined with the age group 65–74 years in the JMDC database.

^#^ Estimated incidence per 100,000 population per year.

**Table 14:** Estimated incidence of Seizure.

Seizure	MDV				JMDC			
	New cases with confirmed or suspected diagnosis in 2019		New cases with confirmed diagnosis in 2019		New cases with confirmed or suspected diagnosis in 2019		New cases with confirmed diagnosis in 2019	
	N	Incidence^#^	N	Incidence^#^	N	Incidence^#^	N	Incidence^#^
Total cases	270,694	214.6	260,238	206.3	329,112	255.0	231,540	179.4
**Gender**								
Male	144,516	235.3	139,216	226.7	158,494	252.4	107,767	171.6
Female	126,179	194.9	121,021	186.9	170,618	257.5	123,773	186.8
**Age (years old)**								
0–11	20,376	169.8	20,982	174.8	33,178	271.3	14,297	116.9
12–17	9,122	138.3	9,503	144.0	22,140	330.3	10,850	161.8
18–24	11,806	133.9	11,708	132.8	20,634	232.9	13,329	150.5
25–44	30,920	105.7	30,094	102.9	50,942	169.8	37,206	124.0
45–64	53,075	157.9	51,335	152.8	70,431	206.2	53,149	155.6
65–74	45,752	263.0	43,572	250.5	131,787	355.3	102,709	276.9
75-*	94,613	511.7	88,287	477.5				
**Literature review data**								
The Japan Epilepsy Society noted that the incidence of epilepsy in developed countries is regarded as 45 per 100,000 population per year [44]. The MHLW estimated the prevalence of epilepsy in Japan to be 5–8 per 1,000 population in Japan [45]								

* As patients above 75 years of age were few, they were combined with the age group 65–74 years in the JMDC database.

^#^ Estimated incidence per 100,000 population per year.

**Table 15:** Estimated incidence of Preterm Labor.

Preterm Labor	MDV				JMDC			
	New cases with confirmed or suspected diagnosis in 2019		New cases with confirmed diagnosis in 2019		New cases with confirmed or suspected diagnosis in 2019		New cases with confirmed diagnosis in 2019	
	N	Incidence^#^	N	Incidence^#^	N	Incidence^#^	N	Incidence^#^
Total cases	132,837	105.3	132,636	105.1	301,000	233.2	297,537	230.6
**Gender**								
Male	20	0.0	20	0.0	18	0.0	18	0.0
Female	132,817	205.1	132,617	204.8	300,982	454.3	297,519	449.0
**Age (years old)**								
0–11	44	0.4	44	0.4	18	0.1	18	0.1
12–17	479	7.3	474	7.2	219	3.3	219	3.3
18–24	12,221	138.6	12,226	138.7	11,128	125.6	11,049	124.7
25–44	119,613	408.8	119,413	408.1	288,887	962.9	285,503	951.6
45–64	479	1.4	479	1.4	748	2.2	748	2.2
65–74	0	0.0	0	0.0	0	0.0	0	0.0
75-*	0	0.0	0	0.0				
**Literature review data**								
National statistics (the vital statistics of the MHLW) reported that the proportion of all births that were preterm in Japan was 5.6% in 2015, 5.8% in 2013 and 4.1% in 1980 [47–49].								

* As patients above 75 years of age were few, they were combined with the age group 65–74 years in the JMDC database.

^#^ Estimated incidence per 100,000 population per year.

**Table 16:** Estimated incidence of Spontaneous Abortion.

Spontaneous Abortion	MDV				JMDC			
	New cases with confirmed or suspected diagnosis in 2019		New cases with confirmed diagnosis in 2019		New cases with confirmed or suspected diagnosis in 2019		New cases with confirmed diagnosis in 2019	
	N	Incidence^#^	N	Incidence^#^	N	Incidence^#^	N	Incidence^#^
Total cases	19,368	15.4	19,129	15.2	82,431	63.9	77,494	60.1
**Gender**								
Male	0	0.0	0	0.0	36	0.1	36	0.1
Female	19,368	29.9	19,129	29.5	82,395	124.4	77,458	116.9
**Age (years old)**								
0–11	0	0.0	0	0.0	17	0.1	0	0.0
12–17	64	1.0	64	1.0	110	1.6	92	1.4
18–24	1,657	18.8	1,628	18.5	3,643	41.1	3,021	34.1
25–44	17,208	58.8	17,002	58.1	76,629	255.4	72,711	242.4
45–64	440	1.3	435	1.3	2,032	5.9	1,670	4.9
65–74*	0	0.0	0	0.0	0	0.0	0	0.0
75-*	0	0.0	0	0.0				
**Literature review data**								
A survey conducted in a hospital in Miyagi, Japan, between 1972 and 1980 reported that the proportion of the incidence of spontaneous abortion among all pregnancies was 9.6% [51]. A survey conducted in Denmark between 1978 and 1992 reported that the proportion of incidence of spontaneous abortion among pregnancies was 13.3% among 12–19 year-olds, 11.1% among 20–24 year-olds, 11.9% among 25–29 year-olds, 15.0% among 30–34 year-olds, 24.6% among 35–39 year-olds, 51.0% among 40–44 year-olds, and 93.4% among those 45 years or older [52]. A survey conducted in Aichi, Japan, between 2007 and 2010 reported that 38.3% of the women with a history of pregnancy had experienced at least one spontaneous abortion [53].								

* As patients above 75 years of age were few, they were combined with the age group 65–74 years in the JMDC database.

^#^ Estimated incidence per 100,000 population per year.

### 1) Bell’s palsy

Bell’s palsy is an acute, monosymptomatic disorder characterized by unilateral peripheral facial paresis (partial) or paralysis (complete) resulting in temporary weakness of facial muscles due to facial nerve dysfunction. Since the causality is unknown, an etiology of exclusion approach is adopted based on anatomical structure, history of viral infection, ischemia, inflammation and cold stimulation responsivity [12]. The estimated incidence of Bell’s palsy in MDV and JMDC were 41.7 and 47.9 cases per 100,000 population per year, respectively, which were higher than the incidence reported in European countries. The age-specific estimate tended to increase with age in both databases (Table 3).

### 2) Guillain-Barre Syndrome

Guillain-Barre Syndrome (GBS) is a typical immune mediated polyradiculoneuropathy characterized by fulminant progression of flaccid muscular weakness and diminished myotatic reflexes thought to be activated by an acute infection. It is estimated that two-thirds of the reported cases resulted from infection caused by several viral species [14]. A predominance in the estimated number and incidence of new cases of the clinical condition was observed in males in comparison with females and the condition also was observed to increase with age in both the MDV and JMDC databases (Table 4).

### 3) Optic neuritis

Optic neuritis is a clinical presentation of idiopathic optic neuropathy affecting the optimal function of the optic nerve that leads to demyelination. The disease manifests in isolation or in a setting with multiple sclerosis or neuromyelitis optical [19]. The gender-based trend analysis revealed that the incidence (N, per 100,000 population per year) was higher in females than males in both databases and according to both Definitions 1 and 2. Elderly patients (over 65 years) had a considerably higher estimated number of cases / incidence (Table 5).

### 4) Idiopathic thrombocytopenic purpura

Idiopathic thrombocytopenic purpura, which is also known as immune mediated thrombocytopenia, is an immunocompromised acquired bleeding disorder occurring as a result of transient or persistent decrease of the platelet count [22]. The estimated number of new cases / incidence based on Definition 1 reflected a slight female preponderance [MDV Definition 1: N = 10,828; 16.7 per 100,000 population per year], [JMDC Definition 1: N = 13,334; 20.1 per 100,000 population per year)]. This condition was notably higher in the elderly population aged over 75 years (Table 6).

### 5) Multiple sclerosis

Multiple sclerosis is the prototype chronic inflammatory pathological condition of autoimmune origin. It is characterized by relapses and remissions of demyelination, gliosis and neuronal loss caused by focal lymphocytic infiltration resulting in severe neurological defects [24]. The estimated number of new cases and incidence was higher in females than males in the two databases. Heterogeneity was high among the age groups which made age-specific trend assessment difficult. Individuals in their 50s and early 60s were observed to be more prone to this condition (Table 7).

### 6) Complex regional pain syndrome

Complex regional pain syndrome, also known as reflex sympathetic dystrophy (RSD), is a post traumatic chronic neurologic disorder. It is characterized by a series of prolonged painful episodes accompanied by sensory, vasomotor, sudomotor and motor functional impairments [27]. Marked female predilection was observed in comparison with males (Table 8). The estimated number and incidence of new cases of the clinical condition were higher in the elderly population (Table 8).

### 7) Hypercoagulable States (Autoimmune)

Hypercoagulable States (Autoimmune) is often termed as antiphospholipid syndrome or antiphospholipid antibody syndrome (APS or APLS), or Hughes syndrome. It is an autoimmune, hypercoagulable state caused by antiphospholipid antibodies that include the lupus anticoagulant, or moderate-high titer anticardiolipin, or anti-β2 Glycoprotein I antibodies. The array of clinical phenotypes leads to arterial and venous thrombosis, microvascular and obstetrical complications such as pre-eclampsia, pregnancy morbidity and fetal demises [29]. A consistently higher (N, per 100,000 population per year) occurrence of the condition was observed in females with increases across successive age groups (Table 9).

### 8) Any Death/Sudden Unexplained Death

It was not possible to estimate the occurrence of sudden unexplained death in the MDV and JMDC databases as these deaths almost exclusively occur outside of healthcare institutions and are therefore not included in the health insurance medical records. Only limited numbers of any unexplained death were available from the MDV and JMDC databases and these numbers are provided in Table 10. In a study from Okinawa, for the period 1 January 1992 to 31 December 1994, the incidence of sudden unexpected death was observed to be 37 per 100,000 population per year (age 20–74 years, males = 51 per 100,000 population per year and females = 23 per 100,000 population per year). Importantly, for 53% of these deaths the cause was undetermined [31]. Further, data of the Utstein registry collected by the Fire and Disaster Management Agency noted that the number of sudden cardiac deaths recorded in Japan was 78,884 in 2019, suggesting an incidence of 62 deaths per 100,000 population per year [32]. Finally, the number of deaths due to any cause in 2019 was 1,381,093 according to the Vital Statistics published by the Ministry of Health, Labour and Welfare [33].

### 9) Acute Transverse Myelitis

Acute Transverse Myelitis (ATM) is a rare etiologically heterogeneous inflammatory subtype of transverse myelopathy. The clinical syndrome has an acute or subacute onset, which on spinal injury affects sensory and motor skills resulting in neurologic deficits [34]. The estimated incidence of confirmed diagnoses of acute transverse myelitis was 0.5–0.6 cases per 100,000 population per year. The incidence in Japan was lower than observed in studies outside of Japan (Table 11).

### 10) Allergic Purpura

Allergic Purpura, also known as Henoch-Schönlein purpura (HSP), is an IgA-mediated systemic microvasculitis condition associated with the accumulation of antibodies in the blood vessels [37]. The pathology is widely observed in the pediatric population. The proportion of female Allergic Purpura cases was higher than male cases in both databases and taking all study groups together there was higher incidence among the age group 0-11(Table 12).

### 11) Anaphylaxis/Shock

Anaphylaxis is a life-threatening and acute multisystemic hypersensitivity condition. The episodes often manifest with a potentially fatal outcome, as the risk of rapid-evolving respiratory collapse is anticipated. Despite the causative factor, expeditious cause determination and treatment is critical [41]. With respect to anaphylaxis, estimated incidence appeared to be higher in men and lower in women for both Definitions 1 and 2 in both databases. The numbers of confirmed or suspected diagnoses were similar to those of confirmed diagnoses (Table 13).

### 12) Seizure

An epileptic seizure is a spontaneous neurological transient array of signs and symptoms resulting from abnormal fulminant or synchronous neuronal activity in the brain [43]. Estimated new cases / incidence varied across different age groups (Table 14).

### 13) Preterm Labor

Preterm birth is one of the crucial obstetrical complications that is attributed to increased risk of perinatal mortality and morbidity. Preterm is a parturition occurring less than 37 completed weeks or 259 days of gestation [46]. There were an estimated 105.3 and 233.2 new cases per 100,000 population per year with a confirmed diagnosis of preterm in the year 2019 in MDV and JMDC, respectively (Table 15).

### 14) Spontaneous abortion

Spontaneous abortion is a pregnancy failure occurring before 20 weeks of gestation as result of natural causative factors [50]. Among the female population in the MDV database, the overall estimated number of new cases / incidence of spontaneous abortion were 19,368 cases (15.4 cases per 100,000 population per year) and 19,129 cases (15.2 cases per 100,000 population per year), respectively. In the JMDC database, the estimated number of new cases / incidence according to Definitions 1 and 2 were 82,431 female cases (63.9 cases per 100,000 population per year) and 77,494 cases (60.1 cases per 100,000 population per year), respectively (Table 16).

## Discussion

Adverse events occurring around the time of vaccination can be misconstrued as a potential outcome attributed to the vaccine in an extensive national vaccination program. This can lead to vaccine hesitancy and resistance to vaccination among the public. Vaccine hesitancy jeopardizes the success of vaccination programs which consequently increases the risk of disease morbidity and mortality. It is critical therefore that steps must be taken to enhance confidence in vaccines to be used in these programs. However, at the same time stringent measures must be put in place to ensure early reporting of adverse events following vaccination in order that these can be investigated thoroughly to determine whether a causal relationship exists between the vaccine and observed clinical conditions. For any adverse event that is less frequent in a vaccinated group than in others, the potential association between the vaccination and the event can also be worthy of further investigation.

The estimates of the number of total cases and incidence were similar for the MDV and JMDC databases for some diseases. In addition, some estimates were similar to those in the scientific literature. However, the interpretation of the data included in this study must consider the limitations that are inherent to the databases which provided the main source of data and the extrapolations that were used to estimate the occurrence of these clinical conditions for the whole of Japan. (1) This retrospective database study only covers reimbursement claims processed within hospitals, which are not specifically recorded for research purposes. Results of laboratory tests or any medical procedures were not available in the current analysis; therefore, we defined the diagnosis only by ICD-10 codes. In addition, incidence of mild and severe clinical conditions could not be differentiated. Further, the presence or absence of other underlying medical conditions was not taken into account. This can lead to either an under- or over-estimate in disease occurrence when data are not entered appropriately into the databases. To capture the appropriate number of cases of disease for Japan, the sets of ICD-10 codes may be different from the ones used in other studies. (2) MDV is strictly a DPC hospital-based database, so there was no traceability of patients when patients switched hospitals. JMDC is a health insurance database, so limited data are available on individuals (and their families) who are not employed in middle- to large-sized companies. (3) Patients included in the databases are also likely to differ. Patients usually visit general practitioners at local clinics first, and afterwards are transferred to or visit large-sized specialized hospitals. For this reason, the JMDC database, as it also includes primary care settings, will inevitably contain more suspected cases of disease as confirmatory diagnoses have not yet been made in the hospital setting. (4) Patients with mild or moderate diseases may visit only primary care settings and will be included in the JMDC database but not in the MDV database, which consists mainly of large-scale specialized hospitals. The data included in these two databases therefore do not precisely represent the disease occurrence in the general Japanese population. The patient groups in these two databases are different, and the data collected emphasizes the need for caution in evaluating the occurrence of clinical conditions, while at the same time providing a range between which the true occurrence may lie. When the estimates of incidence are similar from the two databases, then, these can be considered to be reasonable estimates of the true incidence. (5) Both databases also contain some data entry errors. For example, on a few occasions the gender for patients with spontaneous abortion and preterm labor was recorded as male. (6) Individuals in the MDV database can be lost to follow-up as they move from one healthcare institution to another. For this database it is possible that a single patient can be included as multiple patients if there is a change in hospitals and these hospitals are included in the MDV database. Therefore, this review can only provide a potential range of occurrence of clinical conditions in Japan based on these healthcare databases and the available literature.

In addition, the data collected in this study can also be used to estimate a range for the number of cases of a clinical condition (temporally related to vaccination) that is likely to be observed within 1 day, 1 week or 1 month following vaccination in a hypothetical vaccination program as was suggested by Wang et al. in their publication on the expected number of background disease events during mass immunization in China [8]. Examples of such estimations are provided below in Table 17 based on data from both the MDV and JMDC databases. The calculation uses the estimated incidence of each clinical condition applied to the population of ≥ 65-year-old patients to be vaccinated and assumes that the incidence of the clinical conditions remains constant throughout the year. These expected background rates can be different between people with and without underlying medical conditions. Thus, caution is required when we want to compare patients with a certain underlying medical condition. As noted by Wang and colleagues, even for relatively infrequent clinical conditions, a significant number of observations can be expected in a short period of time because of the substantial number of individuals vaccinated in pandemic situations.

**Table 17:** Expected number of cases for various clinical conditions within different time periods of vaccination for those 65 years and older based on data from the MDV and JMDC databases.

Clinical condition	Expected number of cases* ≥ 65 years old (confirmed or suspected) within 1 day of vaccination		Expected number of cases* ≥ 65 years old (confirmed or suspected) within 1 week of vaccination		Expected number of cases* ≥ 65 years old (confirmed or suspected) within 1 month of vaccination	
	MDV	JMDC	MDV	JMDC	MDV	JMDC
Bell’s palsy	62	78	436	547	1,896	2,375
Guillain-Barre syndrome	8	9	57	61	249	263
Optic Neuritis	21	106	146	744	636	3,233
Idiopathic Thrombocytopenic Purpura	32	28	224	199	972	865
Multiple Sclerosis	7	24	52	171	226	745
Complex Regional Pain Syndrome	5	8	35	58	151	253
Hypercoagulable States (Autoimmune)	35	84	245	585	1,064	2,543
Any Death/Sudden Unexplained Death	1	0	7	0	32	0
Acute Transverse Myelitis	1	2	8	13	33	59
Allergic Purpura	12	25	82	177	356	771
Anaphylaxis/Shock	48	69	338	485	1,469	2,110
Seizure	385	361	2,692	2,527	11,697	10,982

* Rounded to the nearest whole number.

## Conclusion

This is the first report study in Japan, to the authors’ knowledge, to use large-scale, real-world data and published literature to estimate the occurrence of 43 clinical conditions. The primary conclusion of our study is that clinical conditions usually observed after vaccination can also be observed in the situation without vaccination at a certain level of frequency as reported in this study. Some of the clinical conditions will continue to occur following the start of extensive national vaccination programs against COVID-19. Determining whether clinical conditions occurring around the time of vaccination are causally or only temporally related to vaccination will be critical for public health decision makers. This will require in-depth evaluations of the potential for causality. Our study demonstrates that the relationship between vaccination and clinical conditions occurring around the time of vaccination should not be immediately considered as causal. Although in-depth evaluations of causality at the individual case level are essential, causal judgement may be difficult. Alternatively, background occurrence at the population level may provide some additional objective evidence for the evaluation of temporality or causality. The background occurrence reported in this study can be used as one of the elements for the evaluation of the temporal or causal relationships between vaccinations and clinical conditions. Data on the safety profile of COVID-19 vaccines for the general population in Japan is yet to be evaluated. Presently data are available on the wider use of these vaccines in other countries. The authors of this publication are considering following up with a later publication of the safety profile of COVID-19 vaccines when the vaccination program in Japan has advanced further.

## Supporting information

S1 Table All conditions and results in this study. A-AQ: Actual and estimated number of new cases with confirmed diagnosis and with or without suspected diagnosis of various clinical conditions in Japan from 2015 to 2019 based on data from the MDV and JMDC databases.
(XLSX)

S2 Table PICOST (Population, Intervention, Comparator, Outcomes, Study type and Timeframe) table defining inclusion and exclusion criteria for the literature searches. (XLSX)

S3 Table Example search strategy in PubMed for the clinical condition encephalitis. (XLSX)
